# Abiotic environments prevail over plant functional traits in shaping phyllosphere fungal communities of temperate grasslands in China

**DOI:** 10.1093/ismeco/ycaf096

**Published:** 2025-06-23

**Authors:** Shanshan Song, Xian Yang, Rong Tang, Zhiyao Tang

**Affiliations:** State Key Laboratory for Vegetation Structure, Function and Construction (VegLab), Institute of Ecology, and College of Urban and Environmental Sciences, Peking University, 100 Zhongguancun North Street, Haidian District, Beijing 100871, China; State Key Laboratory of Biocontrol, School of Ecology, Sun Yat-sen University, 66 Gongchang Road, Guangming District, Shenzhen 518000, China; State Key Laboratory for Vegetation Structure, Function and Construction (VegLab), Institute of Ecology, and College of Urban and Environmental Sciences, Peking University, 100 Zhongguancun North Street, Haidian District, Beijing 100871, China; State Key Laboratory for Vegetation Structure, Function and Construction (VegLab), Institute of Ecology, and College of Urban and Environmental Sciences, Peking University, 100 Zhongguancun North Street, Haidian District, Beijing 100871, China

**Keywords:** abiotic environments, epiphytic, endophytic, fungi, phyllosphere, plant functional traits

## Abstract

Phyllosphere fungi play critical roles in plant health and ecosystem functioning. While previous studies have explored the diversity and composition of phyllosphere fungal communities in various ecosystems, they have typically focused on either epiphytic or endophytic fungi at single sites and rarely addressed both groups simultaneously across broad environmental gradients. As a result, the relative importance of abiotic environments, plant traits, and dispersal processes in shaping phyllosphere fungal communities remains unclear, particularly with respect to differences between epiphytic and endophytic fungi. We collected 231 leaf samples from nine sites in temperate grasslands of northern China, and explored the effect of abiotic environments and plant traits on the diversity and structure of phyllosphere fungi at broad spatial scales. Our analysis revealed that aridity, poor soil conditions and leaf pH decreased the relative abundance of endophytic saprophytic fungi, and plants with a “fast-growing” strategy promoted the relative abundance of phyllosphere pathogenic fungi. The positive effects of aridity and poor soils on the richness of endophytic fungi were undermined by soil fungal richness, while the richness and Pielou’s evenness of epiphytic fungi was inhibited by the availability of soil resource. Soil organic carbon emerged as a key factor influencing the composition of phyllosphere fungal communities, and dispersal limitation was relatively weak and comparable between endophytic and epiphytic fungi. Our findings provide empirical evidence that abiotic environments prevail over plant traits in structuring phyllosphere fungi at broad spatial scales and reveals assembly patterns that differ from those reported in previous single-site or forest-based studies.

## Introduction

Phyllosphere microorganisms play a pivotal role in facilitating plant nutrient acquisition, disease resistance, and abiotic stress resistance [1–6]. They also influence the dynamics of plant communities and contribute to maintaining ecological stability [7]. Plant leaves provide two distinct habitats for phyllosphere microorganisms: the epiphytic habitat on the leaf surface, characterized by nutrient scarcity and environmental exposure, and the endophytic habitat within the leaf tissues, where apoplast nutrients are compartmentalized and selectively accessible through plant-regulated processes [1, 8, 9]. These contrasting habitat conditions, combined with the influence of host immunity, lead to variations in microbial community diversity and structure between the endophytic and epiphytic habitats [10–12].

The structure and diversity of phyllosphere fungi are influenced by various factors, including climate [13], soil properties [14, 15], and host identity [16, 17]. Given that soil serves as a direct source of phyllosphere microorganisms [18], changes in soil fungal diversity may directly affect phyllosphere fungal communities. The majority of studies have indicated a negative relationship between drought or nutrient stress and soil fungal diversity [19–22], while some demonstrated positive [23] or non-linear [24] relationships. Although it has been proposed that drought stress suppresses dominant fungi on leaves and provides opportunities for more fungi to coexist [25], it remains unclear whether this change is primarily caused by direct environmental stress or indirectly by effects on soil fungal diversity.

Host plant identity and functional traits also play a crucial role in the assembly of phyllosphere fungal communities [16, 26, 27]. In comparison to host plant identity, plant functional traits exert a more direct impact on the structure of phyllosphere microbial communities, as they not only directly reflect differences in plant adaptation to environmental conditions [28, 29], but also capture subtle variations among individuals within the same species [30, 31]. According to the plant functional trait economic spectrum theory, plants can be classified along a continuum from fast-growing acquisitive strategies to slow-growing conservative strategies [32, 33], based on their growth strategies. Some studies have indicated a positive correlation between leaf nutrient content and the size of phyllosphere bacterial communities [34, 35], suggesting that species with a “fast-growing” strategy may support greater fungal diversity in their phyllosphere. Conversely, other studies have suggested that endophytic fungal diversity decreases in leaves with higher nitrogen-to-phosphorus ratios [36] or lower leaf carbon [26]. These contrasting findings highlight the ongoing debate regarding the influence of plant functional traits or nutritional status on the regulation of leaf fungal communities.

Elucidating the effects of environmental stress on the abundance of different microbial taxa is key to understanding changes in microbial diversity [37, 38]. It has been proposed that under environmental stress, plants may prioritize growth over resistance to pathogens, i.e. the growth-defense tradeoff of plants [39]. Normally, pathogen growths faster in resource-rich environments [40]. Tannins and phenolic acids produced by plants as part of their anti-herbivore defense can directly inhibit pathogen growth through antimicrobial activity [41], and may also indirectly alter the composition of the phyllosphere fungal community by changing leaf pH [42]. While plant metabolites play a direct role in regulating leaf pH, it is also influenced by factors such as soil properties and climatic factors [43]. In soil, pH has been widely recognized as a key factor shaping fungal community composition [44–46], with studies showing a positive correlation between pH and the abundance of pathogenic fungi, while saprophytic fungi tend to be more prevalent in lower-pH environments. However, it remains unclear whether a similar relationship between leaf pH and these fungal taxa.

Stochastic dispersal processes, unlike deterministic processes driven by specific biological or environmental factors, refer to the random movement and distribution of fungal spores across different environments [47]. These processes are largely influenced by random unpredictable factors such as water flow, wind, or vector movements, which could transport fungal propagules over varying distances. Fungi with light and small spores and strong reproductive abilities tend to disperse more stochastically than those reliant on specific hosts or habitats [48, 49]. Stochastic dispersal has been shown to have a non-negligible impact on microbial community structure [47, 50]. In particular, soil microbial communities, which serve as an important source of phyllosphere fungi, are increasingly shaped by the stochastic process of dispersal limitation as spatial distance increases [51, 52]. These findings underscore the potential importance of spatial distance in shaping phyllosphere fungal communities across broad environmental gradients.

Although numerous studies have explored the composition and diversity of phyllosphere fungal communities, most have been conducted in agricultural systems or forest ecosystems at a single site [16, 26, 53–57]. These studies typically focus on either endophytic or epiphytic fungi, or analyze the phyllosphere as a whole, emphasizing host identity and fine-scale environmental filtering as key determinants of community structure [26, 54–57]. For example, research in Mediterranean pine–oak mixed forests have shown that plant functional traits such as leaf carbon and water content can strongly influence epiphytic fungal communities [56]. For endophytes, a study in a tropical seasonal evergreen forest highlighted the importance of leaf ambient environment in shaping fungal diversity [57]. Notably, only a few studies have simultaneously examined both endophytic and epiphytic fungi. Among them, one study in a tropical mangrove ecosystem found that plant identity had a stronger influence on endophytic than on epiphytic fungi [16]. Due to the limited spatial extent of these studies—generally spanning only tens to hundreds of meters—they cannot adequately evaluate the influence of broad-scale drivers such as dispersal limitation or climate-related environmental gradients. However, understanding large-scale processes is essential for advancing our knowledge of phyllosphere fungal assembly. In particular, dispersal limitation may exert different effects on endophytic and epiphytic fungi due to their contrasting microhabitats. Moreover, broad-scale variation in climate, soil properties, and plant functional traits may jointly shape fungal diversity and composition in ways that are not detectable by single site studies. Most large-scale studies to date have not distinguished between endophytic and epiphytic fungal communities [13, 36, 58, 59]. For instance, a study across a 100-km soil nutrient gradient in California found that foliar fungal endophyte diversity was shaped by leaf nutrient availability and plant stress conditions [36], but did not examine epiphytic fungi. Two recent studies have assessed both epiphytic and endophytic fungi across large spatial scales, focusing on olive trees in Mediterranean ecosystems [13] and rubber trees in tropical plantations [58]. While these studies revealed distinct drivers of epiphytic and endophytic communities, both of them focused on a single host species and excluded plant functional traits from their analytical frameworks. These limitations leave critical knowledge gaps regarding the mechanistic drivers of phyllosphere fungal assembly processes.

Temperate grasslands in northern China span wide geographic and environmental gradients, with substantial variation in vegetation and abiotic conditions. This system provides an ideal opportunity to examine the relative roles of abiotic environments, host plant traits, and dispersal limitation in shaping phyllosphere fungal communities. In this study, leveraging this natural gradient, we collected 231 leaf samples across nine temperate grassland sites spanning ~1500 km in northern China. We used these samples to investigate the composition and diversity of phyllosphere fungal communities, including both epiphytes and endophytes, and to identify the underlying drivers of their assembly. Specifically, we proposed the following hypotheses. First, plants with a “fast-growing” strategy and high leaf pH may promote the growth of leaf pathogenic fungi while inhibiting the growth of saprophytic fungi (H1), similar to the effect of soil pH on the abundance of soil saprophytic and pathogenic fungi. Second, abiotic environments may indirectly influence the diversity of leaf epiphytic fungi by affecting soil fungal diversity, and may indirectly influence the diversity of leaf endophytic fungi through plant functional traits (H2). Third, dispersal limitation may play a more important role in leaf endophytic fungi than in leaf epiphytic fungi (H3), This may be because endophytic fungi must overcome structural barriers such as the cuticle to colonize internal leaf tissues, which increases their functional dispersal distance. By testing these hypotheses, we aim to fill critical knowledge gaps regarding the mechanisms that shape the diversity and structure of fungal communities in different leaf habitats, and provide a more comprehensive understanding of how abiotic environments and plant functional traits jointly influence phyllosphere fungal communities across environmental gradients.

## Materials and methods

### Study area and sample collection

The study was conducted in July–August of 2022 and 2023 along a transect comprising nine sampling sites (each with an area of two-hectare), i.e. three sites for each of the three different steppe types (desert, typical and meadow steppes), in the temperate grasslands of northern China, with a range of 39.39–49.33°N in latitude, 107.86–120.01°E in longitude, and 640–1603 m in altitude (Table S1). Within each sampling site, we established six 20 m × 20 m sampling plots, with a minimum distance of 30 m between them. Within each plot, three 1 m × 1 m subplots were established along the diagonal at least 10 m apart to investigate species cover and height. We identified species with an average relative cover greater than 5% within each sampling site as dominant species (Table S1).

In each subplot, we selected target plants based on the average height of each dominant species and collected at least 10 g of intact leaves without visible disease symptoms from the aerial parts of plants using sterile gloves, excluding flowers, fruits, or stems. All leaves were sampled during the peak growth period (mid-July to mid-August) of grassland plants within their growing season to ensure consistent leaf maturity and uniform growth stages. In total, we collected 231 leaf samples. These leaves were immediately placed in an ice box and then pre-treated to extract fungal DNA. In addition, we collected leaves from at least three plants of each dominant species within each sampling plot to determine their functional traits. We used a soil drill with a diameter of 7 cm to randomly collect three surface soil samples (top 10 cm) from each subplot, which were mixed and used to determine soil properties and extract fungal DNA.

### Climate and soil properties

According to local meteorological observations, the mean annual temperature (MAT) in the study area ranges from 0.2°C to 7.1°C, and the mean annual precipitation (MAP) ranges from 229 mm to 438 mm (Table S1). We obtained the potential evapotranspiration (PET) data from the CGIAR-CSI database [60], and calculated the moisture index (MI) as the ratio of MAP to PET. We used the water extraction method to determine soil pH with a soil/water volume ratio of 1:5, the molybdenum-antimony spectrophotometry method to measure soil total phosphorus (TP), the molybdenum-antimony colorimetry method to determine soil available phosphorus (AP) [61], and an elemental analyzer (FLASHEA 112 Series, Thermo Electron, United States) to measure soil organic carbon (SOC) and total nitrogen (TN). We measured soil ammonium nitrogen (NH_4_^+^-N) and nitrate nitrogen (NO_3_^−^-N) using Ultraviolet spectrophotometry method extracted with potassium chloride [62].

### Plant functional traits

We measured a total of six plant functional traits, i.e. leaf nitrogen content (LN), leaf phosphorus content (LP), specific leaf area (SLA), leaf dry matter content (LDMC), leaf pH, and plant height. Firstly, we soaked the leaf samples in ordinary distilled water for at least 24 h to obtain their saturated fresh weight, then we used an Epson V39 scanner to measure the leaf surface area, and finally we dried the scanned leaves in an oven at 65°C for 48 h to obtain their dry weight. We used an elemental analyzer (FLASHEA 112 Series, Thermo Electron, United States) to determine LN, and the molybdenum-antimony colorimetric method to measure LP [63]. We calculated SLA as the ratio of leaf area to corresponding dry weight, and LDMC as the ratio of dry weight to saturated fresh weight. We used the water extraction method to determine leaf pH with a soil/leaf volume ratio of 1:10 [64].

### Pre-treatment of leaves

We pre-treated the leaves following the method described by Yang et al. (2023) [12]. First, we weighed 4 g of fresh leaves and placed them in a centrifuge tube containing 50 ml of sterile potassium dihydrogen phosphate buffer (K_2_HPO_4_ + KH_2_PO_4_, 0.1 M, pH = 7.5) [12]. We then vortexed the samples at 4000 rpm for 5 mins. After that, we transferred the buffer to a new centrifuge tube and centrifuged it at 12 000 g for 10 mins. After removing the supernatant, we used the remaining material in the centrifuge tube to extract the DNA of leaf epiphytic fungi.

We sterilized the leaf surface before extracting the DNA from leaf endophytic fungi. We first weighed 4 g of fresh leaves and placed them in a centrifuge tube containing 50 ml of sterile water, then vortexed the samples at 3000 RPM for one minute. We soaked the leaves in 1% sodium hypochlorite for one minute, then in 75% ethanol for one minute (repeated twice), and finally in sterile water for one minute (repeated twice). We ground the sterilized leaves under liquid nitrogen freezing conditions using a sterile mortar and pestle to extract endophytic fungal DNA.

### Fungal DNA extraction from leaves and soil

We extracted and purified the DNA from the precipitate in the centrifuge tubes and ground leaves according to the instructions of the ALFA-SEQ Advanced Soil DNA Kit, and then determined the concentration and purity of the DNA samples using a NanoDrop One spectrophotometer (Thermo Fisher Scientific, MA, USA). We conducted Polymerase chain reaction (PCR) amplification of the ITS1–2 region of leaf fungi using specific primers BD-ITS1F (CTTGGTCATTTAGAGGAAGTAA) and ITS2-2043R (GCTGCGTTCTTCATCGATGC) with 2 × Taq Master Mix (Quick Load) (Novoprotein Scientific Inc. Suzhou, China). We extracted and purified the DNA from the topsoil according to the instructions of the PowerSoil DNA Kit, and after determining its concentration and purity, we performed PCR amplification of the ITS2 region of soil fungi using specific primers gITS7 (GTGARTCATCGARTCTTTG) and ITS4 (TCCTCCGCTTATTGATATGC). We detected the lengths and concentrations of PCR products using 1% agarose gel electrophoresis and GeneTools analysis software (Version4.03.05.0, SynGene), respectively, and then mixed the PCR products according to the principle of equal mass, and finally purified the PCR products using the E.Z.N.A. Gel Extraction Kit (Omega, USA). We performed Double terminal PE250 sequencing based on the Illumina Nova 6000 platform (Guangdong Magigene Biotechnology Co., Ltd. Guangzhou, China).

### Bioinformatics analysis

First, we used the CUTAADAPT (https://github.com/marcelm/cutadapt/) to remove primers and obtain paired-end clean reads after quality control. We then used the USEARCH-fastq_mergepairs (http://www.drive5.com/usearch/) to obtain the original spliced sequence (raw tags) by filtering out unqualified tags. The prediction parameters included a minimum overlap length of 16 bp and a maximum allowed mismatch of 5 bp in the overlap region of the spliced sequences. We used the fastp package (version 0.14.1, https://github.com/OpenGene/fastp) to crop the raw tag data by sliding the window quality to obtain valid spliced fragments (clean tags), then used the DADA2 plugin in QIIME2 to denoise the raw sequencing reads and infer the amplicon sequence variants (ASVs), and finally used the sintax command in USEARCH to annotate ASVs based on the UNITE database (version 8.0, https://unite.ut.ee/).

### Statistical analysis

Prior to the diversity analyses, we rarefied the sequence counts to 11 064 for leaf epiphytic and endophytic fungal samples and 17 955 for soil fungal samples to ensure consistency in the sampling effort (Fig. S1). Given the differences in sequencing primers and dilution levels between soil and phyllosphere fungi, the subsequent analysis focuses solely on soil fungal diversity as an explanatory variable, without directly comparing the differences between phyllosphere and soil fungi. We used the *vegan* package to calculate the diversity of phyllosphere and soil fungi, including ASV richness and Pielou’s evenness [65]. We conducted a genus level functional prediction for the ASVs table based on the FungalTraits database [66] classifying fungi into pathogenic, saprotrophic and mycorrhizal fungi based on ecological strategies and functions [67]. All data were standardized prior to analysis (mean = 0, *SD* = 1).

To test the first and second hypotheses, we first performed principal component analysis (PCA) to reduce the dimensionality of the abiotic environmental variables and plant functional traits, respectively. We then used linear mixed-effects models to explore the effect of scores on the first and second principal component axes on the diversity of phyllosphere fungi and the relative abundance of different taxa, with plots grouped within sites as random intercepts. To further elucidate the direct and indirect effects of abiotic environments, plant functional traits, and the diversity of soil fungi (Div_Soil_) on the diversity of leaf endophytic and epiphytic fungi, we performed piecewise structural equation modelling (SEM) using the *piecewiseSEM* package [68], using the same random structure. We constructed an initial SEM with all possible pathways (Fig. S2), and then sequentially removed non-significant pathways to obtain the final model. The final model was chosen based on two criteria: the *P* values obtained from Fisher’s C test and chi-square test were greater than 0.05, and the minimization of the Akaike’s Information Criterion (AIC) to achieve an optimal balance between model complexity and fit. Since our primary focus is on ASV richness, which reflects the number of taxa present and is widely used in ecological studies to assess microbial diversity patterns, we performed SEM analysis based on ASV richness.

To test the third hypothesis, we performed Hellinger standardization on the original ASVs counts [69, 70], and then calculated Bray-Curtis dissimilarities (*β*-diversity) among leaf endophytic, epiphytic, and soil fungal samples separately. We calculated spatial distances between samples based on longitude and latitude using the *geosphere* package [71], and assessed differences in climate, soil, and plant functional traits between samples using Euclidean distance with the *vegan* package [64]. We calculated the phylogenetic distance between species using the “cophenetic” function from the *ape* package [72], based on a phylogenetic tree constructed with the “phylo.maker” function in the V.PhyloMaker2 package [73]. The backbone of this tree is the “GBOTB.extended.WP.tre” file, which primarily follows the seed plant phylogeny from Smith & Brown [74] while incorporating the pteridophyte (fern) branch structure from Zanne et al. [75]. We further explored the influence of abiotic environments (climatic factors and soil properties), spatial distance, soil fungal community composition (*β*-diversity_Soil_), and plant attributes (plant functional traits, Phy_Plant_: species phylogenetic distance) on the composition of leaf endophytic and epiphytic fungal communities (*β*-diversity), using the *ecodist* package through multiple regression on dissimilarity matrices (MRM) [76]. We first standardized all variables using the “stdize” function from the *MuMin* package [77], then constructed the full MRM model and performed stepwise regression analysis using the “step” function of the *stats* package to select the final MRM model based on the lowest AIC. Finally, we performed variance partitioning analysis (VPA) to quantify the relative contributions of the selected factors in the final MRM model [65].

To ascertain whether the composition of phyllosphere fungal community deviates from neutral expectations, we calculated the *β*-deviation by comparing the observed Bray-Curtis values with those generated by the null model [78]. Specifically, we constructed 999 simulated null communities by randomly assigning ASVs to each sample, while maintaining constant ASV richness in each sample and the occurrence frequency of each ASV across all samples [79].

All analyses were performed in R version 4.3.3 [80].

## Results

### Composition and diversity of phyllosphere fungi

In total, 14 325 385 and 16 956 003 high-quality fungal sequences were identified from 223 endophytic and 231 epiphytic samples, respectively. These sequences were further grouped into 4145 and 7324 fungal ASVs, respectively.

**Figure 1:**
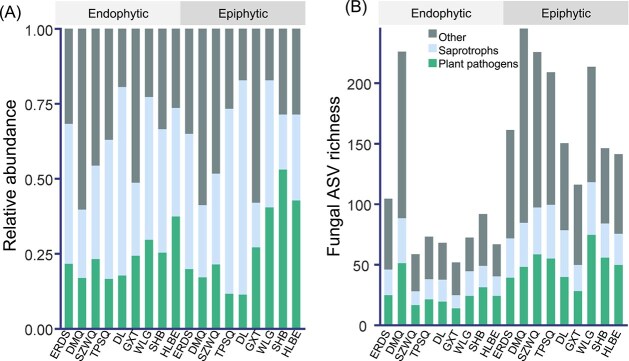
Composition and diversity of fungal communities in the phyllosphere. (A) Relative abundance of different fungal functional groups. (B) Diversity of different fungal functional groups.

Ascomycota and Basidiomycota were the dominant phyla in the phyllosphere fungal communities, with mean relative abundance ranging from 45.6% to 88.5% (mean 74.0%) for Ascomycota and 4.5% to 51.5% (mean = 21.1%) for Basidiomycota, across different sites (Fig. S3A). Plant pathogens and saprotrophs were the dominant functional groups, with mean relative abundance ranging from 14.9% to 71.5% (mean 38.7%) for plant pathogens and 11.3% to 53.0% (mean = 25.4%) for saprotrophs, across different sites (Fig. 1A).

Ascomycota and Basidiomycota together accounted for 86.7% to 93.2% (mean = 90.9%) of the total fungal ASVs at different sites (Fig. S3B). Plant pathogens and saprotrophs contributed 34.5% to 61.4% (mean = 49.5%) of the total fungal ASVs across the sites (Fig. 1B).

### Effects of abiotic environments and plant functional traits on the relative abundance of the dominant functional groups

**Figure 2:**
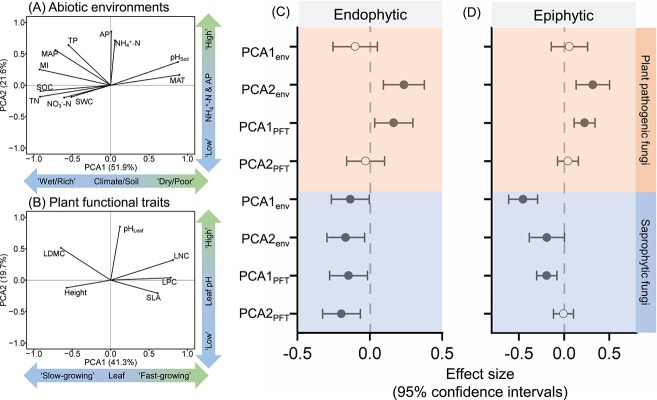
Principal component analysis (PCA) of abiotic environments (A) and plant functional traits (B), and linear mixed-effect models examining the effects of scores on the first and second principal component axes on the relative abundance of plant pathogenic and saprophytic fungi (C, D). Solid and open circles represent significant (*P* < .05) and non-significant (*P* > .05) effects, respectively. Error bars indicate the standard error of the estimates. PCA1_env_: Scores on the first axis of the principal component analysis of abiotic environments; PCA2_env_: Scores on the second axis of the principal component analysis of abiotic environments; PCA1_PFT_: Scores on the first axis of the principal component analysis of plant functional traits; PCA2_PFT_: Scores on the second axis of the principal component analysis of plant functional traits; MAT: mean annual temperature (°C); MAP: mean annual precipitation (mm); MI: moisture index; pH_soil_: soil pH; SWC: soil water content (%); SOC: soil organic carbon (g/kg); TN: total nitrogen (g/kg); NH_4_^+^-N: ammonium nitrogen (mg/kg); NO_3_^-^-N: nitrate nitrogen (mg/kg); TP: total phosphorus (g/kg); AP: available phosphorus (mg/kg); pH_Leaf_: leaf pH; LDMC: leaf dry matter content (%); SLA: specific leaf area (cm^2^mg^-1^); LNC: leaf nitrogen content (%); LPC: leaf phosphorous content (%).

The PCA of abiotic environments revealed that the first axis (PCA1_env_) captured the climate and soil fertility gradient, ranging from “wet/rich” to “dry/poor”, and the second axis (PCA2_env_) reflected the gradient of soil NH_4_^+^-N and AP nutrient availability, ranging from “low” to “high” (Fig. 2A; Table S2). Linear mixed-effects models showed that the relative abundance of leaf endophytic and epiphytic saprophytic fungi decreased with both PCA1_env_ and PCA2_env_. The relative abundance of leaf endophytic and epiphytic plant pathogenic fungi increased with PCA2_env_ (Fig. 2C & 2D). For dominant fungal phyla, the relative abundance of leaf endophytic and epiphytic Ascomycota increased with PCA1_env_, while that of Basidiomycota decreased with PCA1_env_ (Fig. S4).

In the PCA of plant functional traits, the first axis (PCA1_PFT_) represented the conservation gradient of resource acquisition, ranging from “slow-growing” to “fast-growing” strategies (Fig. 2B; Table S2), and the second axis (PCA2_PFT_) represented the leaf pH gradient, ranging from “low” to “high”. Linear mixed-effects models showed that the relative abundance of leaf endophytic and epiphytic plant pathogenic fungi increased, while that of leaf endophytic and epiphytic saprophytic fungi decreased with PCA1_PFT_. In comparison, the relative abundance of leaf endophytic saprophytic fungi decreased with PCA2_PFT_ (Fig. 2C & 2D). Regarding dominant fungal phyla, the relative abundance of leaf epiphytic Ascomycota increased, while that of leaf epiphytic Basidiomycota decreased with PCA1_PFT_ (Fig. S4).

### Factors influencing the diversity of phyllosphere fungi

**Figure 3:**
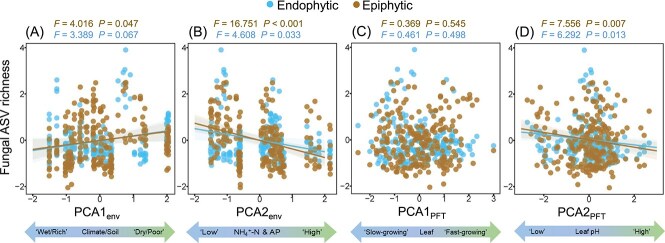
Linear mixed-effects models examining the effects of abiotic environments and plant functional traits principal component scores on the ASV richness of leaf endophytic and epiphytic fungi. The scatter shown in the figure is the standardized data (mean = 0, *SD* = 1). The solid line represents the significant (*P* < .05) or marginal significant (*P* < .1) slope value for the linear mixed-effects model, and the corresponding shaded area represents the fitted 95% confidence interval.

Linear mixed-effects models showed that the ASV richness of both epiphytic and endophytic fungi increased with PCA1_env_ (Fig. 3A), but decreased with PCA2_env_ (Fig. 3B). The ASV richness of leaf endophytic and epiphytic fungi showed non-significant trend with PCA1_PFT_ (Fig. 3C), while both decreased with PCA2_PFT_ (Fig. 3D). Pielou’s evenness of leaf epiphytic fungi increased with PCA1_env_ (Fig. S5a), but decreased with PCA2_env_ (Fig. S5b).

**Figure 4:**
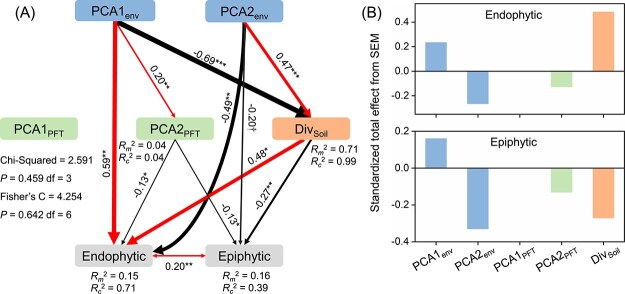
Piecewise SEM showing the direct and indirect effects of abiotic environments (PCA1_env_ and PCA2_env_), plant functional traits (PCA1_PFT_ and PCA2_PFT_) and soil fungal ASV richness (Div_Soil_) on the ASV richness of leaf endophytic and epiphytic fungi. The numbers on the arrows represent standardized path coefficients. The arrow thickness is proportional to the magnitude of these path coefficients. The significance levels are denoted as follows: † *P* < .1, * *P* < .05, ** *P* < .01, and *** *P* < .001. Two-way arrows indicate significant correlations between variables. *R_m_^2^* and *R_c_^2^* represent the marginal and conditional *R^2^* values of each dependent variable in the model.

The SEM results showed that PCA1_env_ had a direct positive effect on the ASV richness of leaf endophytic fungi, but an indirect negative effect on the ASV richness of leaf endophytic fungi through PCA2_PFT_ and Div_Soil_ (Fig. 4A). PCA2_env_ had a direct negative effect on the ASV richness of leaf endophytic and epiphytic fungi, and an indirect negative effect on the ASV richness of leaf epiphytic fungi through Div_Soil_ (Fig. 4A). Standardized total effects from the SEM indicated that the ASV richness of leaf endophytic fungi was primarily influenced by Div_Soil_, and the ASV richness of leaf epiphytic fungi was primarily determined by PCA2_env_ (Fig. 4B).

### Factors influencing the composition of phyllosphere fungal community

**Figure 5:**
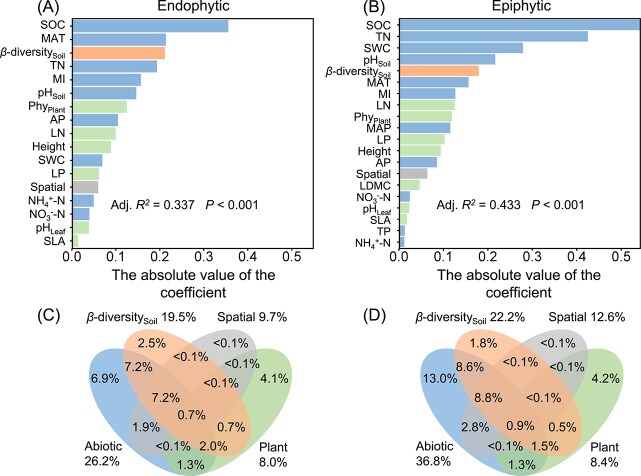
Multiple regression on dissimilarity matrices (MRM) analysis of the effects of selected abiotic environments (climatic factors and soil properties), plant attributes (plant functional traits, Phy_Plant_: Species phylogenetic distance), spatial distance, and soil fungal community composition (*β*-diversity_Soil_) on the composition of leaf endophytic and epiphytic fungal communities (A & B), and variation partitioning for the composition of leaf endophytic and epiphytic fungal communities based on MRM (C & D).

Multiple regression on dissimilarity matrices (MRM) showed that the selected variables explained 33% and 43% of the overall variation in the composition of leaf endophytic and epiphytic fungal community, respectively, with SOC having the most significant impact (Fig. 5A & 5B). VPA based on MRM indicated that the relative contribution of abiotic environments, both pure and total fractions, to the composition of endophytic and epiphytic fungi was greater than that of the composition of soil fungal community, plant attributes, and spatial distance (Fig. 5C & 5D).

## Discussion

Our study examined the influence of abiotic environments and plant functional traits on the diversity and structure of phyllosphere fungi in temperate grasslands, where herbaceous plants dominate and both plant hosts and environmental conditions vary greatly across spatial scales. This study advances previous work on phyllosphere fungal communities in three key ways. First, our broad-scale sampling across temperate grasslands, encompassing wide environmental gradients, allowed us to capture spatial and environmental drivers not addressed in single site studies. Second, we explicitly assessed the relative importance of abiotic conditions, plant functional traits, and dispersal processes, and found that abiotic factors prevail over plant functional traits overall. Third, by simultaneously examining both endophytic and epiphytic communities, we found similarly low dispersal limitation in both groups, contrasting with previous findings from forest ecosystems. Together, these results offer a more comprehensive understanding of phyllosphere fungal community assembly across spatial scales. The specific results are discussed in detail below.

### Response of the abundance of different fungal taxa to abiotic environments and plant functional traits

Similar to previous findings that soil pH suppressed soil saprophytic fungi [44, 81], we observed that soil pH was also detrimental to the abundance of phyllosphere saprophytic fungi (Fig. 2). The effect of leaf pH on the abundance of leaf endophytic saprophytic fungi mirrored the influence of soil pH, supporting our hypothesis (H1). This suggests that leaf pH may be related to the decomposition rate of leaves after they wither by affecting the growth of saprophytic fungi. However, the response of saprophytic fungi to abiotic environments in the phyllosphere differed from that in soil [82], with water and nutrient limitations hindering the growth and reproduction of phyllosphere saprophytic fungi. This may be due to the lower overall litter production in arid regions.

The survival and reproduction of plant pathogens are highly dependent on carbon sources provided by living plants [83]. Consistent with the results of previous nutrient addition experiments [45, 84], both soil nutrient availability and plant nutrient content acted consistently positive effect on the abundance of phyllosphere pathogenic fungi. Rich soil nutrients promote leaf growth and nutrient content, creating favorable conditions for the proliferation of leaf pathogenic fungi. Additionally, according to the plant growth-defense strategy trade-off [39], plants with a “fast-growing” strategy and high nutrient content are more susceptible to pathogenic fungi. Ascomycetes, as opportunists, thrive in nutrient-rich environments [85]. The abundance of leaf epiphytic Ascomycetes was significantly promoted by both soil resource availability and plant nutrient content. This reveals a coupled relationship between the survival strategies of fungi in leaf epiphytic habitats and the growth strategies of plants.

### Response of the diversity of phyllosphere fungi to abiotic environments and plant functional traits

Consistent with previous studies in the temperate grasslands of northern China [86, 87], we found that aridity reduces soil fungal richness (Fig. 4). While many studies consider soil as a repository for phyllosphere microorganisms [9, 51], we found a promoting effect of soil fungal richness on the richness of leaf endophytic fungi, but not leaf epiphytic fungi (Fig. 4), which is inconsistent with our initial hypothesis (H2). A possible explanation is that increased soil fungal richness may benefit plant root health and nutrient uptake [88], which in turn indirectly supporting the survival and reproductive capacity of leaf endophytic fungi. Another possible explanation is that a diverse microbial community in the soil may enhance leaf immunity through mechanisms such as sufficient nutrient provisioning [89], immune priming [90], or other indirect effects. By strengthening plant defense responses, these mechanisms could suppress pathogen activity in the endosphere [91], potentially create a more favorable environment for a greater diversity of rare commensal taxa. However, in regions with high soil fungal richness, a more diverse pool of fungi may disperse to the leaf surface, potentially increasing competition for space and resources among epiphytic fungi. While moderate microbial exchange might enhance diversity, excessive competition and priority effects could limit the establishment of certain taxa, potentially constraining overall epiphytic fungal richness. Alternatively, soil fungal richness and epiphytic fungal diversity may be independently shaped by other environmental factors that influence both communities.

Aridity and nutrient-poor soils can directly promote the richness of leaf endophytic fungi to some extent (Fig. 4), potentially because certain endophytic fungi are more resilient and adaptable under environmental stress [92]. Moreover, aridity stress might reduce the abundance of certain dominant taxa, thus creating ecological space for a greater variety of species to grow and reproduce [29, 36]. However, the reduction in soil fungal richness undermined this positive effect. Consistent with previous work on the effect of nutrient addition on soil fungal diversity [93, 94], we found that soil available nitrogen and phosphorus nutrient levels exert a detrimental influence on leaf epiphytic fungal richness and evenness.

Previous studies conducted in a subalpine timberline ecotone and a series of coastal terraces have suggested a correlation between plant functional traits, or the fast-slow resource acquisition strategy, and leaf endophytic fungal diversity [26, 36]. However, no such relationship was observed in our study, suggesting that the factors influencing leaf endophytic fungal diversity are more complex than we had assumed and may involve the interaction of multiple factors. Particularly noteworthy is our finding that leaf pH, an important plant functional trait, is a critical factor in explaining phyllosphere fungal richness. Soil pH had a positive effect on phyllosphere fungal richness, which is consistent with previous studies on soil fungi [95, 96]. Surprisingly, leaf pH had an opposite effect, further emphasizing the complexity of mechanisms that affect the richness leaf phyllosphere fungi, which are jointly shaped by internal physiological processes and external environmental factors in plants. While leaf pH significantly influenced the richness of phyllosphere fungi, their effects on evenness were not significant. This suggests that leaf pH may primarily shape the presence or absence of fungal taxa rather than their relative abundance.

### Mechanisms of the composition of fungal communities in the phyllosphere

Our results differ from previous studies conducted within single forest plots, such as those conducted in a subtropical forest [17] and Mediterranean pine–oak mixed forest [56], which identified host traits as the dominant factor in fungal community assembly. In contrast, across our multi-site sampling spanning a broad environmental gradient in temperate grasslands, abiotic factors, particularly SOC, emerged as the primary determinants of phyllosphere fungal community composition. This finding is consistent with large-scale studies on soil microorganisms [97], and suggests that when variation in climate and edaphic conditions is substantial, environmental filtering may override the influence of host plant traits in driving the community assembly of phyllosphere fungi.

We also examined the relative importance of spatial distance and found that it explained a comparable proportion of variation in the composition of leaf epiphytic fungi (12.6%) and endophytic fungi (9.7%). Although we initially hypothesized that endophytic fungi would be more constrained by dispersal (H3), our results revealed similar spatial patterns for epiphytic and endophytic fungi, with environmental factors explaining more variation than spatial distance for both groups. This result contrasts with findings from a large-scale study of field-grown rubber trees, where spatial distance exerted a greater influence than environmental factors on both endophytic and epiphytic fungal communities [58]. One possible explanation is that herbaceous plants in our study systems typically have thinner leaves with simpler anatomical barriers, which may may lower the barrier for endophyte colonization and enhance their dispersal. Additionally, the open and continuous vegetation structure of temperate grasslands may promote aerial dispersal of fungal propagules more effectively than forest canopies [98]. Together, these results suggest that dispersal limitation plays a weaker role in shaping phyllosphere fungal communities in grasslands compared to forests—a pattern analogous to conclusions from previous soil microbial research [99, 100].

In addition, we found that the differences in community composition and *β*-deviation of leaf endophytic fungi were reduced in aridity and nutrient-poor soil environments (Fig. S6). This may be due to plants relying more on symbiotic relationship with endophytic fungi for essential nutrients and water under environmental stress [101]. Different plant individuals may depend on similar fungal taxa, and this convergent dependence leads to an increase in the similarity of endophytic fungal community composition across plants.

## Conclusion

In this study, we explore the impact of abiotic environments and plant functional traits on the diversity and composition of phyllosphere fungal communities in temperate grasslands at broad spatial scales. We derived three novel findings. First, aridity, poor soil conditions and leaf pH decreased the relative abundance of leaf endophytic saprophytic fungi, and plants with a “fast-growing” strategy promoted the relative abundance of phyllosphere pathogenic fungi. Second, the positive effects of aridity and poor soils on the richness of leaf endophytic fungi were undermined by soil fungal richness, while the richness and Pielou’s evenness of leaf epiphytic fungi was inhibited by the availability of soil resource. Third, soil organic carbon emerged as a key factor influencing phyllosphere fungal community composition, and dispersal limitation appeared relatively weak and comparable between endophytic and epiphytic communities. These findings provide comprehensive empirical evidence that abiotic environments prevail over plant functional traits in shaping phyllosphere fungal communities at broad spatial scales, thereby filling a key research gap in understanding large-scale assembly mechanisms of these microbial communities. We also provide the first large-scale empirical comparison of endophytic and epiphytic fungi in temperate grasslands, revealing assembly mechanisms that differ from those reported in forest ecosystems. These insights advance our understanding of microbial community assembly in plant-associated habitats and offer a foundation for grassland management strategies informed by microbial communities.

While our findings provide valuable insights into the factors shaping phyllosphere fungal communities, certain limitations should be acknowledged. Our functional classification relies on the FungalTraits database, which assigns functions at the genus level. While this is a widely used approach in microbial ecology, it does not capture species-specific functional variation, potentially limiting the resolution of our findings. Additionally, since our research is based on temperate grassland ecosystems, the observed patterns may not be directly applicable to other ecosystems, such as managed cropping systems, ecosystems with different climatic conditions, or those with distinct plant community compositions, where dispersal and assembly mechanisms may differ. Future research across diverse ecosystems is needed to further assess the generality of these findings.

## Supplementary Material

Supplement_material_ycaf096

## Data Availability

Raw Illumina sequence data are deposited in the Sequence Read Archive (SRA) of the National Center for Biotechnology Information (Endophytic: PRJNA1193242; Epiphytic: PRJNA1193225; Soil: PRJNA1193319). Plant functional traits, environmental data and codes in this study are available online from the Dryad Digital Repository (https://doi.org/10.5061/dryad.gtht76hx1).
