# Supplementary material for: Abiotic environments prevail over plant functional traits in shaping phyllosphere fungal communities of temperate grasslands in China
Source: ISME Commun. 2025 Jun 23;5(1):ycaf096. doi: 10.1093/ismeco/ycaf096 (PMC12276378; doi:10.1093/ismeco/ycaf096)
Supplement: Supplement_material_ycaf096 [file supplement_material_ycaf096.docx]

Table S1. Information on sampling sites from west to east in temperate grasslands of northern China. MAT: mean annual temperature; MAP: mean annual precipitation; MI: moisture index

| Location | Type | Lat. (°N) | Lon. (°E) | Ele. (m) | MAT (℃) | MAP (mm) | MI | Sampling species |
| --- | --- | --- | --- | --- | --- | --- | --- | --- |
| ERDS | Desert | 39.39 | 107.86 | 1346 | 7.1 | 259 | 0.17 | *Artemisia ordosica*, *Psammochloa villosa*, *Cleistogenes songorica*, *Stipa breviflora* |
| DMQ | Desert | 41.35 | 111.22 | 1603 | 2.5 | 283 | 0.20 | *Stipa krylovii*, *Leymus chinensis* |
| SZWQ | Desert | 41.78 | 111.90 | 1442 | 3.6 | 229 | 0.16 | *Stipa breviflora*, *Allium polyrhizum*, *Convolvulus ammannii*, *Artemisia frigida* |
| TPSQ | Typical | 42.11 | 115.50 | 1372 | 1.6 | 407 | 0.31 | *Stipa krylovii*, *Cleistogenes squarrosa*, *Convolvulus ammannii*, *Artemisia frigida* |
| DL | Typical | 42.05 | 116.29 | 1317 | 2.1 | 386 | 0.31 | *Leymus chinensis*, *Potentilla acaulis*, *Lespedeza bicolor*, *Artemisia frigida*, *Stipa krylovii* |
| GXT | Typical | 44.15 | 116.34 | 1104 | 4.1 | 350 | 0.28 | *Stipa grandis*, *Anemarrhena asphodeloides*, *Leymus chinensis*, *Cleistogenes squarrosa* |
| SHB | Meadow | 42.31 | 117.28 | 1544 | 0.2 | 438 | 0.41 | *Sanguisorba officinalis*, *Medicago ruthenica*, *Potentilla longifolia*, *Carex korshinskyi*, *Leymus chinensis*, *Stipa grandis* |
| WLG | Meadow | 46.26 | 119.26 | 959 | 0.3 | 321 | 0.30 | *Potentilla longifolia*, *Sibbaldianthe bifurca, Medicago ruthenica, Klasea centauroides,* *Thalictrum aquilegiifolium, Leymus chinensis* |
| HLBE | Meadow | 49.33 | 120.01 | 640 | 1.1 | 333 | 0.35 | *Stipa baicalensis*, *Leymus chinensis*, *Klasea centauroides*, *Bupleurum smithii*, *Astragalus laxmannii*, *Carex korshinskyi* |

Table S2. The standardized variable loadings for PC1 and PC2. PC, principal component.

| Variables | *df* | PCA1_env._ | PCA2_env._ | Variables | *df* | PCA1_PFT_ | PCA2_PFT_ |
| --- | --- | --- | --- | --- | --- | --- | --- |
| MAT | 231 | 0.91 | 0.17 | SLA | 231 | 0.62 | -0.21 |
| MAP | 231 | -0.73 | 0.57 | LDMC | 231 | -0.66 | 0.52 |
| MI | 231 | -0.94 | 0.26 | LPC | 231 | 0.79 | 0.04 |
| pH_Soil_ | 231 | 0.89 | 0.38 | LNC | 231 | 0.82 | 0.32 |
| TN | 231 | -0.93 | -0.18 | pH_Leaf_ | 231 | 0.12 | 0.86 |
| TP | 231 | -0.56 | 0.65 | Height | 231 | -0.58 | -0.12 |
| AP | 231 | 0.01 | 0.87 |  |  |  |  |
| NH_4_^+^-N | 231 | 0.06 | 0.73 |  |  |  |  |
| NO_3_^-^-N | 231 | -0.62 | -0.20 |  |  |  |  |
| SOC | 231 | -0.92 | -0.10 |  |  |  |  |
| SWC | 231 | -0.52 | -0.19 |  |  |  |  |


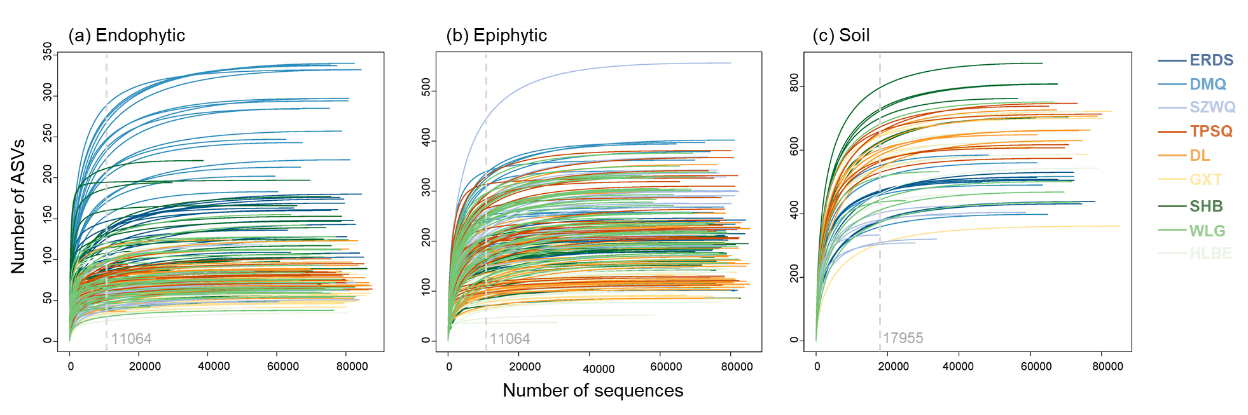


Figure S1. Rarefaction curves of leaf endophytic (a), epiphytic (b), and soil (c) fungi. The vertical dashed line represents the lowest sequence number of the original sample, based on which all samples were diluted to control for the sampling effort.


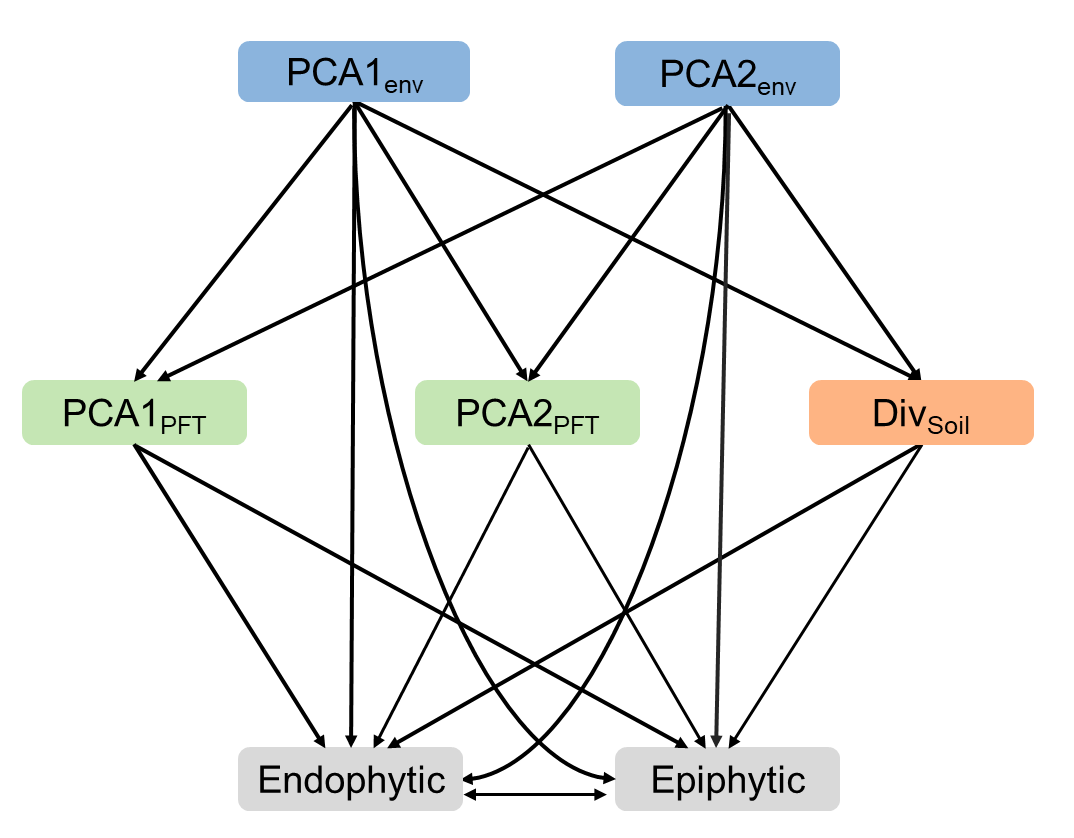


Figure S2. The initial structural equation modeling of the direct and indirect effects of abiotic environments (PCA1_env_ and PCA2_env_), plant functional traits (PCA1_PFT_ and PCA2_PFT_) and the ASV richness of soil fungi (Div_Soil_) on the ASV richness of leaf endophytic and epiphytic fungi.


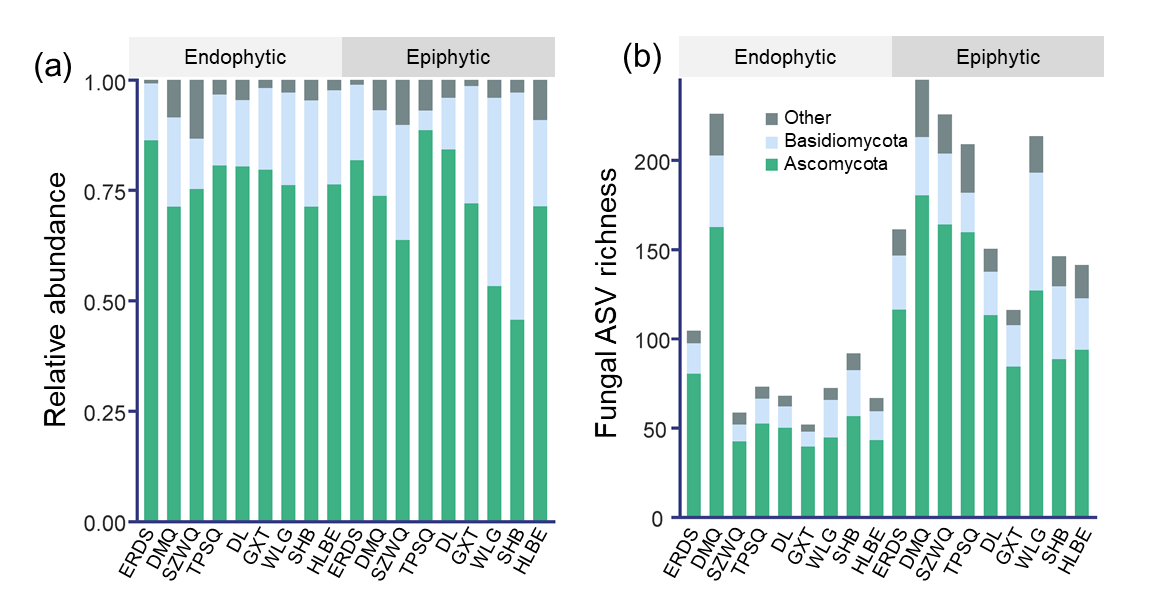


Figure S3. Composition and diversity of the fungal community in the leaf phyllosphere. (a), relative abundance of fungi at the phylum level. (b), diversity of fungi at the phylum level.


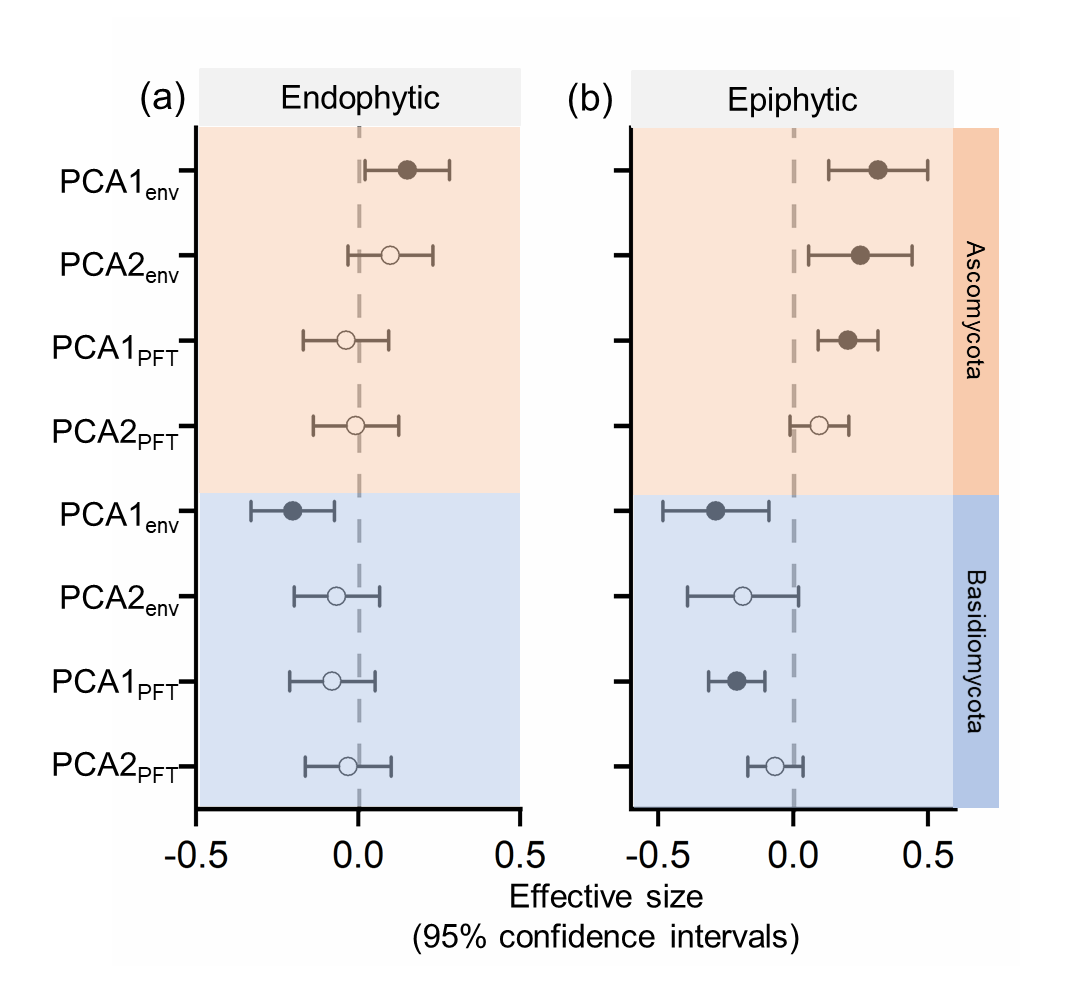


Figure S4. The linear mixed-effect models of abiotic environments and plant functional traits principal component scores on the relative abundance of Ascomycota and Basidiomycota. Solid and open circles represent significant (*P* < 0.05) and non-significant (*P* > 0.05) effects, respectively. Error bars indicate the standard error of the estimates.


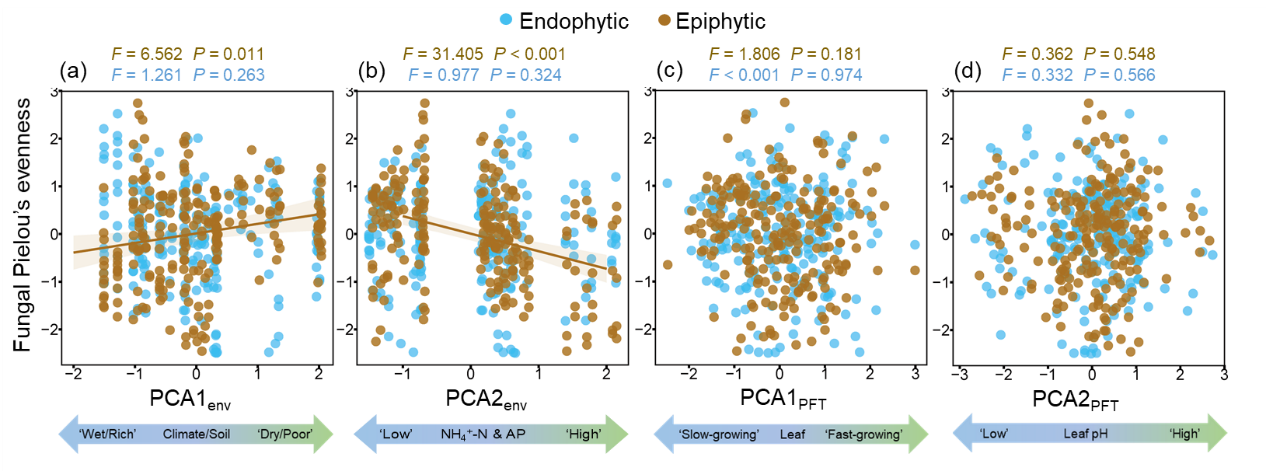


Figure S5. Linear mixed-effects models of abiotic environments and plant functional traits principal component scores on Pielou’s evenness of leaf endophytic and epiphytic fungi. The scatter shown in the figure is the standardized data (mean = 0, *SD* = 1). The solid line represents significant slope value (*P* < 0.05) for the linear mixed-effects model, and the corresponding shaded area represents the fitted 95% confidence interval.


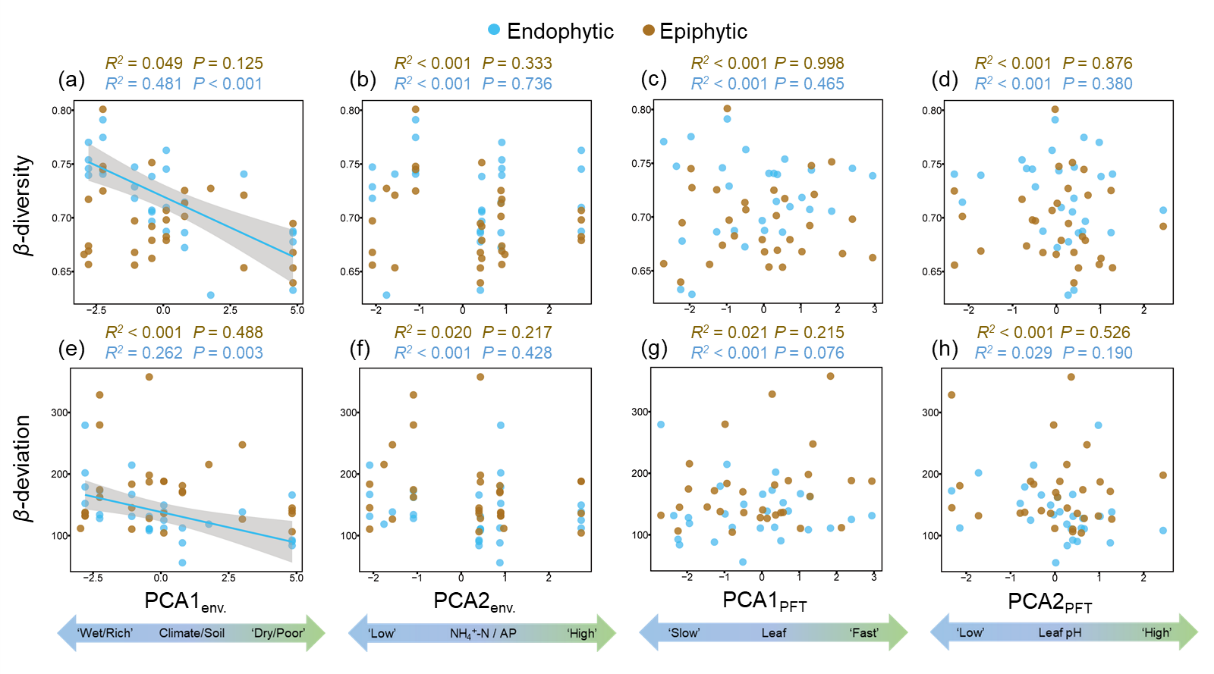


Figure S6. *β*-diversity and *β*-deviation of leaf endophytic and epiphytic fungal communities at the species level in each site, and their relationship with abiotic environments and plant functional traits principal component scores. The *β*-deviation was obtained by comparing the observed Bray-Curtis values with the Bray-Curtis values generated by the null models. ASVs were randomly assigned to each sample to obtain the null community, while ensuring that ASV richness in each sample and the occurrence frequency of each ASV in all samples remained constant. In this way, 999 simulated ‘null communities’ were generated.
